# Extracellular Vesicle-Associated Tissue Factor Activity in Prostate Cancer Patients with Disseminated Intravascular Coagulation

**DOI:** 10.3390/cancers13071487

**Published:** 2021-03-24

**Authors:** Lena Hell, Thomas Däullary, Vanessa Burghart, Lisa-Marie Mauracher, Ella Grilz, Bernhard Moser, Gero Kramer, Johannes A. Schmid, Cihan Ay, Ingrid Pabinger, Johannes Thaler

**Affiliations:** 1 Clinical Division of Haematology and Haemostaseology, Department of Medicine I, Medical University of Vienna, 1090 Vienna, Austria; lena.hell@meduniwien.ac.at (L.H.); thomas.daeullary@uni-wuerzburg.de (T.D.); vanessa.burghart@meduniwien.ac.at (V.B.); n0811056@students.meduniwien.ac.at (L.-M.M.); ella.grilz@meduniwien.ac.at (E.G.); cihan.ay@meduniwien.ac.at (C.A.); ingrid.pabinger@meduniwien.ac.at (I.P.); 2 Center for Physiology and Pharmacology, Institute of Physiology, Medical University of Vienna, 1090 Vienna, Austria; bernhard.moser@meduniwien.ac.at (B.M.); johannes.schmid@meduniwien.ac.at (J.A.S.); 3 Department of Urology, Medical University of Vienna, 1090 Vienna, Austria; gero.kramer@meduniwien.ac.at

**Keywords:** prostate cancer, disseminated intravascular coagulation, tissue factor, extracellular vesicles, peripheral blood mononuclear cells, platelets

## Abstract

**Simple Summary:**

Disseminated intravascular coagulation (DIC) may occur in patients with advanced prostate cancer. In the present study, we detected elevated extracellular vesicle (EV)-associated tissue factor (TF) activity in the plasma of prostate cancer patients with DIC compared with matched prostate cancer patients without DIC and healthy individuals. TF-exposing EVs from DIC patients were highly coagulant in a clotting assay. In in vitro co-culture experiments, EV-TF activity was increased by interactions between a TF-exposing prostate cancer cell line (DU145), peripheral blood mononuclear cells (PBMCs), and platelets. Data from this study contribute to the understanding of the pathogenesis of prostate cancer-related DIC.

**Abstract:**

Patients with advanced prostate cancer may develop fulminant disseminated intravascular coagulation (DIC). Circulating extracellular vesicles (EVs)-exposing tissue factor (TF), the initiator of the coagulation cascade, may play an important role. We included 7 prostate cancer patients with DIC, 10 age- and stage-matched cancer controls without DIC, and 10 age-matched healthy male individuals. EV-TF activity was highly elevated in prostate cancer patients with DIC (11.40 pg/mL; range: 4.34–27.06) compared with prostate cancer patients without DIC (0.09 pg/mL; range: 0.00–0.30, *p* = 0.001) and healthy controls (0.18 pg/mL; range: 0.09–0.54; *p* = 0.001). Only EVs from patients with DIC reduced fibrin clot formation time of pooled plasma in a TF-dependent manner. Next, we performed in vitro co-culture experiments including EVs derived from a prostate cancer cell line with high (DU145) and low (LNCaP) TF expression, peripheral blood mononuclear cells (PBMCs), and platelets. Co-incubation of DU145 EVs with PBMCs and platelets significantly increased EV-TF activity in conditioned medium and induced TF activity on monocytes. No such effects were seen in co-culture experiments with LNCaP EVs. In conclusion, the findings indicate that elevated EV-TF activity plays a role in the development of prostate-cancer-related DIC and may result from interactions between tumor-derived EVs, monocytes, and platelets.

## 1. Introduction

Disseminated intravascular coagulation (DIC) is an “acquired syndrome characterized by the intravascular activation of coagulation with loss of localization arising from different causes” [1]. The first case of DIC in advanced prostate cancer was reported by Rudolf Jürgens almost a century ago [2]. Half a century later, Erwin Deutsch hypothesized that “thromboplastic material which triggers thrombin formation is secreted by circulating tumor cells,” but he did not provide more detail [3]. Since then, numerous reports from prostate cancer patients with DIC have been published [4,5,6,7,8,9]. However, more systematic investigations are lacking, and the pathophysiologic mechanisms underlying DIC in prostate cancer largely remain to be elucidated.

Tissue factor (TF) is the transmembrane receptor of coagulation factor VII. Under physiological conditions, TF is absent from the circulation and only upon vascular damage does subendothelial TF become exposed to blood, which initiates the coagulation cascade. Cancer cells [10] and activated monocytes [11,12] have the ability to shed highly coagulant TF-bearing extracellular vesicles (EVs) from their surface into the circulation. Consistently, increased EV-associated TF (EV-TF) activity was found in patients with different malignancies and acute venous thromboembolism (VTE) [13] or DIC [14]. Elevated levels of monocyte-derived TF-exposing EVs were found in patients with acute myocardial infarction [15], VTE [16], and sepsis [17]. 

We speculated that TF-exposing EVs play a role in prostate-cancer-related DIC. Therefore, we collected plasma samples from prostate cancer patients with DIC, matched prostate cancer patients without DIC, and matched healthy individuals, and performed coagulation experiments. We also performed in vitro co-culture experiments with prostate cancer cell lines, peripheral blood mononuclear cells (PBMCs), and platelets to investigate the impact of cellular interactions on TF activity.

## 2. Materials and Methods

### 2.1. Patients and Controls

The protocol of this study was approved by the local Ethics Committee in accordance with the Declaration of Helsinki. Between 2009 and 2019, patients with DIC and metastatic prostate cancer (*n* = 7), age- and stage-matched prostate cancer patients without DIC (*n* = 10), and age-matched healthy controls (*n* = 10) were included. The presence of overt DIC was evaluated with the overt DIC score of the International Society on Thrombosis and Haemostasis (ISTH). According to this score, ≥5 points indicates ongoing overt DIC (scoring: platelet count > 100G/L = 0 points; <100 = 1; <50 = 2; D-dimer increase: none = 0; moderate = 2; strong = 3; prothrombin time decrease in percent: none = 0; moderate = 2; strong = 3; and fibrinogen >1.0 g/L = 0; <1.0 g/L = 1). Cancer patients ≥ 18 years of age with an ISTH overt DIC score ≥ 5 were considered for inclusion in this study. At study inclusion, informed consent was obtained, patients underwent a structured interview on their medical history, data on tumor histology and stage were collected, and a blood sample was drawn.

Matched male control subjects were from the same geographic region and ethnic background and were neither related to the patients nor to each other.

Venous blood samples were drawn into citrate vacuum tubes (Vacuette, Greiner-Bio One, Kremsmünster, Austria) by an atraumatic and sterile antecubital venipuncture on the day of study entry. The citrated blood was centrifuged at 3000× *g* for 10 min to obtain platelet-poor plasma. The centrifugation of each sample was performed within 1 h after blood sampling and the freezing of each sample within 1 h after centrifugation. Plasma aliquots were stored at −80 °C until measurements were performed in series. 

### 2.2. Routine Laboratory Parameters

Routine laboratory parameters were determined in the central laboratory of the General Hospital Vienna according to protocols that are implemented in routine clinical practice (http://www.kimcl.at/ (accessed on 23 March 2021)). 

### 2.3. Extracellular Vesicle Isolation

Isolation of EVs was performed according to a previously published protocol [18]. Briefly, EVs were pelleted twice by high-speed centrifugation at 18,000× *g* for 20 min at 4 °C. After each centrifugation step, the supernatant was removed carefully except for 50 μL containing the EV pellet, which was washed twice with filtered HBSA (137 mM NaCl, 5.38 mM KCl, 5.55 mM glucose, 10 mM HEPES, and 0.1% bovine serum albumin (BSA), pH 7.4) to reduce contamination through plasma proteins. After the second centrifugation step, 50 μL of the EV pellet was re-suspended in 200 μL of HBSA and vortexed.

### 2.4. Extracellular-Vesicle-Associated Tissue Factor Activity and Cell-Based Tissue Factor Activity 

To analyze EV-associated TF (EV-TF) activity, an established chromogenic factor (F) Xa generation assay was performed as reported previously [18]. This assay was modified to measure cell-bound TF activity in cell culture experiments. Cells were washed twice with HBSA and layered with 50 μL HBSA. Cells or EVs were isolated, incubated with either an antibody for human TF (HTF-1, 500 mg/mL, BD Healthcare, East Ruthford, NJ, USA) or a control antibody (mouse IgG, 500 mg/mL, Sigma, Kawaski, Japan), and measurements were performed in 96-well plates. In the next step, HBSA containing FVIIa, FX (both Enzyme Research, Indianapolis, IN, USA), and CaCl_2_ were added to each sample and the mixture was incubated for 2 h at 37 °C. Then FXa generation was stopped by adding HBSA containing EDTA. Afterward, the chromogenic substrate (Pefachrome, Pentapharm, Basel, Switzerland) for FXa was added, absorbance was measured at 405 nm, and the TF-dependent FXa generation was calculated. The cell-based TF activity assays were performed in triplicates, and the EV-associated TF activity assays in duplicates.

### 2.5. Clotting Assay

Fibrin generation was measured after adding isolated EVs from the plasma or cell culture supernatant (10 μL) to the EV-depleted normal-pooled plasma (centrifuged for 1 h at 150,000× *g*; 60 μL) in the absence or presence of an anti-TF antibody (HTF-1, 500 mg/mL, BD Healthcare, Franklin Lakes, NJ, USA) or control antibody (mouse IgG, 500 mg/mL, Sigma). Fibrin clot generation was initiated by adding CaCl_2_ (20 mM, 20 μL). The plasma turbidity curve reflecting the time until the onset of clot formation was recorded with a Multiskan Spectrum microplate reader (Thermo Scientific Inc., Waltham, MA, USA) at a wavelength of 405 nm. Each measurement was performed in duplicate. 

### 2.6. Cell Culture

**Figure 1:**
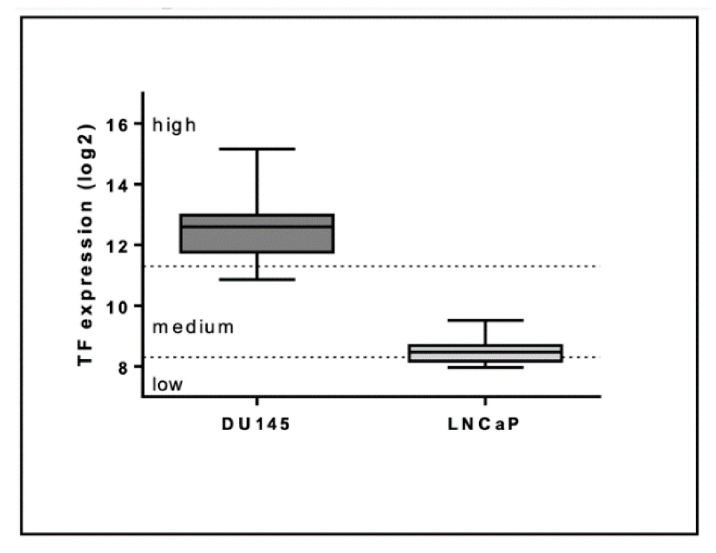
Tissue factor (TF) expression of DU145 and LNCaP cells was assessed with the Genevestigator platform (https://genevestigator.com/ (accessed on 23 March 2021)). The whiskers represent minimum and maximum values, the boxes the 25–75% percentile, and the lines the median values (*n* = 57 for DU145 and *n* = 26 for LNCaP). Low, medium, and high expression are defined as set by the Genevestigator platform.

The TF high DU145 prostate cancer cell line was a gift from Matthias Unseld (Medical University of Vienna, Comprehensive Cancer Center), and the TF low LNCaP prostate cancer cell line was a gift from Bastian Hösel (Institute of Physiology, Center for Physiology and Pharmacology, Medical University of Vienna; Figure 1). Both cell lines (50 cell/μL) were cultured in a humidified incubator at 5% CO_2_ and 37 °C in RPMI-1640 complemented with 1% FCS (Gibco, Waltham, MA, USA), 1% penicillin/streptomycin (Gibco), and HEPES (20 mM) (Gibco) for 24 h. Then, the EV-containing supernatant, i.e., the conditioned medium, was collected, centrifuged at 500× *g* for 5 min at room temperature (RT) to remove remaining cells and debris, and used for subsequent co-culturing.

For co-culture experiments, venous blood from 5 healthy male individuals was collected into citrate vacuum tubes (Vacuette, Greiner-Bio One, Kremsmünster, Austria), centrifuged at 120× *g* for 20 min to gain platelet-rich plasma, which was transferred into new, sterile falcon tubes and centrifuged to pellet platelets. The platelets were then washed twice, counted, and briefly stored until needed. The taken amount of platelet-rich plasma was substituted with sterile PBS and whole blood was used to isolate PBMCs via the Ficoll-Hypaque gradient (Ficol-Paque PLUS, GE Healthcare). PBMCs were washed twice, counted, and briefly stored until used. PBMCs (1500 cells/μL) and platelets (150 × 10^3^ cells/μL) were then co-cultured with the conditioned medium derived from tumor cells (DU145 and LNCaP) for 4 h at 37 °C. Afterward, the supernatant was collected and stored at −80 °C for further analysis, and cells were analyzed immediately. Five experiments using DU145 cells were performed with cells from 5 different healthy donors. Three experiments using LNCaP cells were performed with cells from 3 different donors. 

### 2.7. Flow Cytometry

Cells from co-cultures were harvested by adding Accutase (Sigma) and incubated for 30 min at 37 °C. Harvested cells were fixed in a fixation buffer (2% paraformaldehyde in 1× PBS) and blocked with a flow cytometry buffer (2% BSA in 1× PBS). PBMCs were stained with anti-CD14 BV650 (BioLegend) and anti-TF FITC (Sekisui Diagnostics, Tokyio, Japan). DNA was stained using Syto 41 Blue (ThermoFisher). After staining, the cells were washed and resuspended in the flow cytometry buffer and stored in the dark until analysis. Flow cytometric data were obtained at a CytoFlex S (Beckman Coulter, Brea, CA, USA) using CytExpert v1.2.11.0 software (Beckman Coulter). The flow cytometric data were analyzed with FlowJo v10 software (TreeStar, Ashland, OR, USA).

### 2.8. Statistical Analysis

Continuous variables from clinical data were described by the median and range. The differences between the two groups (prostate cancer patients with DIC versus prostate cancer patients without DIC, prostate cancer patients with DIC versus healthy individuals, and prostate cancer patients without DIC versus healthy controls) were determined using the Wilcoxon–Mann–Whitney U-Test, and Bonferroni-corrected *p*-values were used to indicate statistical significance. Experimental data are described by the mean and standard deviation (SD) and were analyzed using a paired *T*-test or a one-way repeated-measured ANOVA. The analyses were performed with GraphPad Prism v6 (GraphPad Software, La Jolla, CA, USA) and SPSS Statistics v24 (IBM, Armonk, NY, USA).

## 3. Results

### 3.1. Patient Characteristics and Routine Laboratory Parameters

Clinical characteristics and laboratory parameters of prostate cancer patients with DIC (*n* = 7), age- and sex-matched prostate cancer patients without DIC (*n* = 10), and healthy male controls (*n* = 10) are presented in Table 1; Table 2. The D-dimer levels were high in prostate cancer patients with DIC, slightly elevated in prostate cancer patients without DIC, and within the normal range in healthy individuals. Thrombocyte count and fibrinogen levels were below the normal range in patients with prostate cancer and DIC and within the normal range in all other study participants (Table 2).

### 3.2. Patient Case

**Figure 2:**
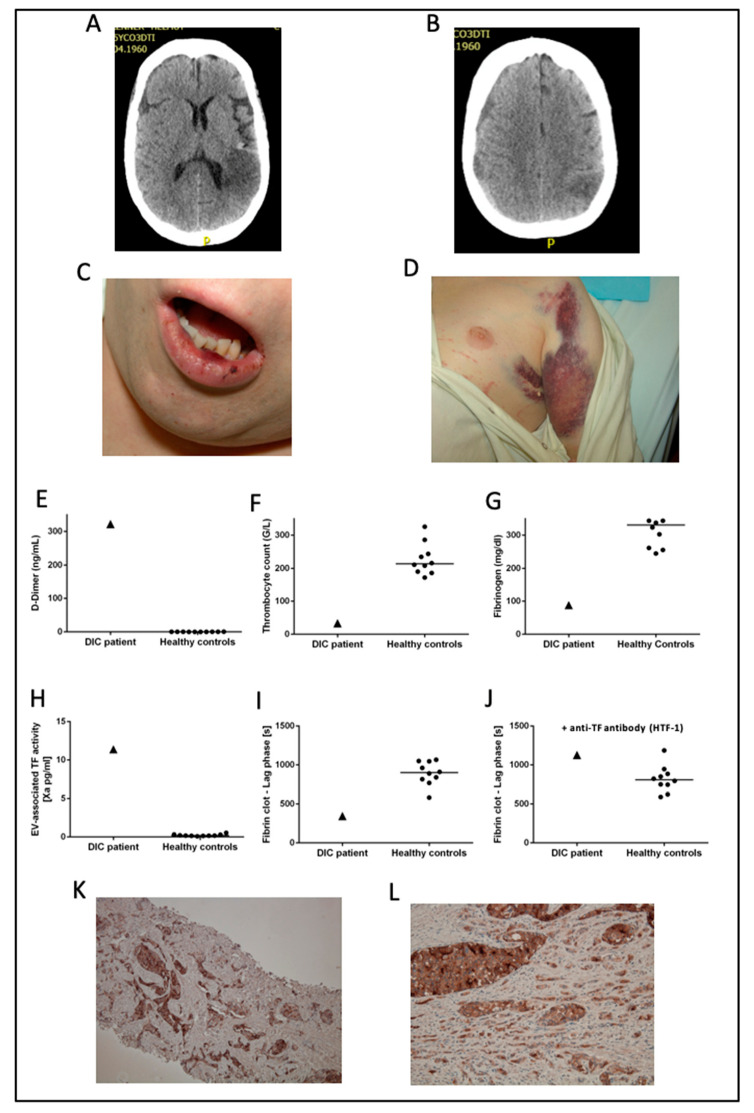
A case of a prostate cancer patient with overt DIC included in the present study. In (**A**,**B**) cerebral computed tomography scans of the patient are shown. Mucosal oozing and disseminated subcutaneous hematomas are shown in (**C**,**D**). In (**E**–**J**), each triangle represents a single measurement of the DIC patient, each dot represents a single measurement of a healthy control subject, and lines represent the median in healthy controls. In (**K**), immunohistochemical staining for TF in a prostate punch biopsy obtained at diagnosis is shown. In (**L**), staining for TF in the prostate obtained post mortem is shown. Abbreviations: ISTH—International Society on Thrombosis and Haemostasis; EV—extracellular vesicle. (**K**): ×100, (**L**): ×400.

As an example, we provide detailed information on a prostate cancer patient with overt DIC who was included in the present study (Figure 2). A male 51 year old patient with castration-resistant metastatic prostate cancer was admitted to the hospital because he had developed a cerebral seizure. A cerebral computed tomography scan revealed a concomitant stroke in the area of the left posterior cerebral artery (Figure 2A) and a subdural hematoma of the right hemisphere (Figure 2B). In the clinical examination, mucosal oozing (Figure 2C) and disseminated subcutaneous hematomas were apparent (Figure 2D). Laboratory analyses revealed deflected coagulation parameters including an extremely elevated D-dimer (Figure 1E), low thrombocyte count (Figure 1F), and low fibrinogen (Figure 2G) corresponding to an ISTH DIC score of six, indicating the presence of overt DIC. In experimental laboratory analysis, elevated plasma EV-TF activity was detected (Figure 2H). Adding isolated EVs from the DIC patient shortened the clotting time of EV-depleted normal-pooled plasma in a TF-dependent manner (Figure 2I,J). The immunohistochemical staining for TF was strong in a prostate punch biopsy (obtained at diagnosis, which was three years before the occurrence of DIC, Figure 2K) as well as in the prostate obtained post mortem (Figure 2L). As the prostate cancer was progressive (with multiple osteolytic and osteoblastic metastases) despite heavy pre-treatment, it was decided to abstain from further causal anti-cancer therapy. As the progressive prostate cancer was regarded as the underlying cause of DIC, no substantial improvement was expected. For the symptomatic hemostatic treatment, fibrinogen and platelets were repeatedly substituted. Eight days after hospitalization, the patient deteriorated and died. 

### 3.3. EV-TF Activity

**Figure 3:**
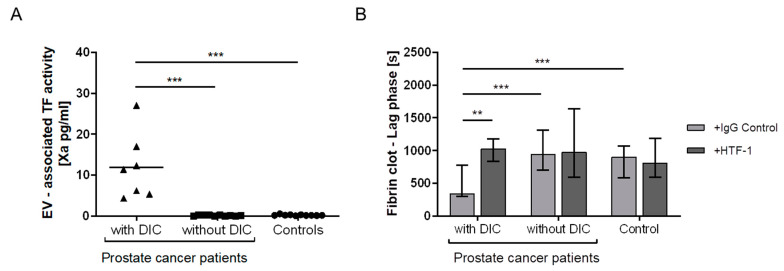
Analysis of the EV-TF activity and EV-TF-dependent fibrin clot formation. EV-TF activity was elevated in patients with prostate cancer and DIC compared with patients with prostate cancer without DIC and healthy controls (**A**). Only EVs from prostate cancer patients with DIC shortened the clotting time of EV-depleted normal-pooled plasma in a TF-dependent manner (**B**). In Figure 3A, each triangle or dot represents a measurement of a single patient, and the line represents the median. Figure 2B shows a bar blot representing the median and whiskers representing the range. (** *p* ≤ 0.01, *** *p* ≤ 0.001, Wilcoxon–Mann–Whitney U-Test). Abbreviations: TF—tissue factor; HTF-1—human tissue factor 1 blocking antibody.

EV-TF activity was significantly elevated in patients with prostate cancer and overt DIC compared to prostate cancer patients without DIC and healthy controls (Table 2, Figure 3A).

### 3.4. Plasma Clotting

EVs from prostate cancer patients with DIC significantly shortened the clotting time of EV-depleted normal-pooled plasma compared with EVs from prostate cancer patients without DIC and EVs from healthy individuals (Table 2, Figure 3B). The clotting time of pooled plasma was prolonged by adding anti-TF antibody only in experiments with EVs from DIC patients (*p* = 0.003), and no significant prolongation of clotting time was observed after adding EVs from prostate cancer patients without DIC (*p* = 0.734) and EVs from healthy individuals (*p* = 0.199, Table 2, Figure 3B). 

### 3.5. Co-Incubation of Conditioned Media from Prostate Cancer Cell Lines with PBMCs and Platelets

**Figure 4:**
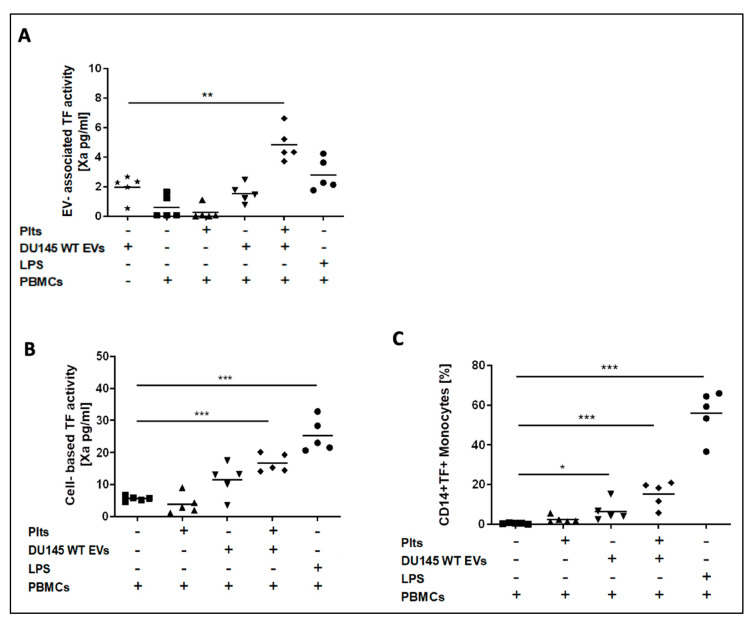
Co-incubation of conditioned media from TF-exposing DU145 prostate cancer cells with peripheral blood mononuclear cells (PBMCs) and platelets. EV-TF activity was detectable in conditioned media from the DU145 cell line. Low EV-TF activity was found in conditioned media from PBMCs alone and PBMCs that were co-incubated with platelets. Co-incubation of PBMCs with DU145 EVs did not significantly increase EV-TF activity. EV-TF activity significantly increased when DU145 EVs, PBMCs, and platelets were co-incubated (**A**). Cell-based TF activity was detectable but low on unstimulated PBMCs and PBMCs that were co-incubated with platelets. Cell-based TF activity significantly increased by co-incubation of PBMCs with EVs from DU145 cells and further increased when platelets were added (**B**). By applying flow cytometry, we found that only monocytes (CD 14+) expose TF but not lymphocytes (CD14−) after co-incubation with DU145-derived EVs and platelets (**C**). Cell-based TF activity (pg/mL) was determined in triplicate. Each dot represents a single measurement and the line represents the mean. (* *p* ≤ 0.05, ** *p* ≤ 0.01, *** *p* ≤ 0.001, paired comparison for repeated measurements). Abbreviations: LPS—lipopolysaccharide.

EV-TF activity was detectable in conditioned media from the TF-exposing DU145 cell line (mean: 1.96 ± 0.84 pg/mL). Low EV-TF activity was found in conditioned media from PBMCs alone (mean: 0.61 ± 0.77 pg/mL) and PBMCs that were co-incubated with platelets (mean: 0.25 ± 0.48 pg/mL). Co-incubation of PBMCs with DU145 EVs did not increase EV-TF activity (mean: 1.55 ± 0.63 pg/mL; *p* = 0.328). EV-TF activity was significantly increased by co-incubation of DU145 EVs, PBMCs, and platelets (mean: 4.84 ± 1.13 pg/mL, *p* = 0.003; Figure 4A).

Cell-based TF activity was detectable on PBMCs alone (mean: 5.65 ± 0.79 pg/mL) and on PBMCs that were co-cultured with platelets (mean: 3.88 ± 3.13 pg/mL), presumably due to mild in vitro activation. Co-incubation of PBMCs with conditioned media from DU145 cells increased the PBMC cell membrane-associated TF activity (mean: 11.51 ± 5.15 pg/mL, *p* = 0.052). A significant further increase in the PBMC cell membrane-associated TF activity was observed when platelets were also added (mean: 16.68 ± 2.83 pg/mL, *p* = 0.001; Figure 4B). 

From the flow cytometric analysis of PBMCs, monocytes (CD14+) exposed TF after co-incubation with DU145 EVs and platelets (Figure 4C). Lymphocytes did not expose TF.

**Figure 5:**
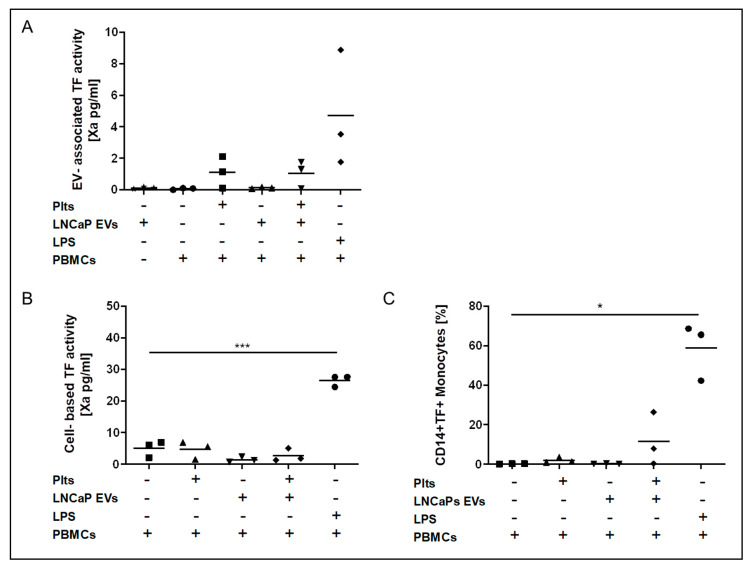
Co-incubation of conditioned media from TF-low LNCaP prostate cancer cells with PBMCs and platelets. EV-associated TF activity (**A**), cell-based TF activity (**B**), and TF expression on monocytes (**C**) did not change significantly when PBMCs were co-incubated with EV from LNCaP cells compared to PBMCs alone in the presence and absence of platelets. Each dot represents a single measurement, and the line represents the mean. (* *p* < 0.05, *** *p* ≤ 0.001, paired comparison for repeated measurements).

In conditioned media from TF, low LNCaP cells EV-TF activity was not detectable, and LNCaP conditioned media did not induce EV-TF activity after co-incubation with PBMCs in the absence and presence of platelets (Figure 5A). Cell-membrane-associated TF activity of PBMCs in the presence and absence of platelets was not induced by conditioned media from LNCaP cells (Figure 5B).

## 4. Discussion

Dysregulation of the hemostatic system may lead to overt DIC in patients with prostate cancer [5]. In the present study, we detected elevated EV-TF activity in the plasma of prostate cancer patients with overt DIC. TF-exposing EVs from DIC patients were highly coagulant in a clotting assay. TF-exposing EVs were not detectable in the plasma of matched prostate cancer patients without DIC and healthy individuals.

To gain further insights into the pathogenesis of prostate cancer-related DIC, we performed in vitro cell co-culture experiments. Such investigations have not been previously performed in tumor models to the best of our knowledge. First, we investigated conditioned media from two prostate cancer cell lines. EV-TF activity was only detectable in conditioned media from the TF-exposing DU145 prostate cancer cell line, indicating that TF-exposing EVs are directly released from these cells. No EV-TF activity was detected in the conditioned media from the TF low LNCaP prostate cancer cell line. 

We speculated that the cells of the tumor-host may also contribute to elevated EV-TF activity in prostate cancer-related DIC and therefore performed co-culture experiments. Co-incubation of DU145 conditioned media with PBMCs from healthy donors did not significantly increase EV-TF activity but induced TF activity on the surface of PBMCs. Performing flow cytometric analysis of PBMCs, we found that TF expression was induced on monocytes but not on lymphocytes, which is in line with previous reports [12,19]. We can only speculate whether TF was transferred from DU145 cells to monocytes or whether TF synthesis was induced on monocytes. On one hand, it was demonstrated that myeloid cells can incorporate cancer-cell-derived EVs [20]. On the other hand, it was also shown that cytokines induce TF expression on monocytes [21]. Tumor-derived cytokines could also play a role in the upregulation of TF on monocytes as it was shown that DU145 cells release IL-6 [22], which is a potent inducer of TF expression on monocytes [23]. 

We also investigated the role of platelets in prostate cancer-related DIC. We did not detect TF activity on platelets per se and no EV-TF activity in conditioned medium from platelets, which is in line with previous reports [24,25]. However, co-incubation of platelets with EVs derived from DU145 cells increased EV-TF activity and cell-based TF activity on monocytes. Hence, our data point to platelet–monocyte interactions that lead to an upregulation or transfer of TF. Platelet–monocyte aggregates could play an important role as they occur upon activation of platelets and result in binding through P-selectin to PSGL-1, which was shown to induce TF expression on monocytes [26,27]. A low platelet count due to platelet activation and consumption as well as increased soluble P-selectin can be found in patients with thrombotic disorders, including DIC and malignancy [28]. Furthermore, cancer cells or cancer-cell-derived EVs are able to activate platelets, induce platelet aggregation, and upregulate P-selectin expression [27]. Platelets shed procoagulant EVs upon activation. Such platelet-derived EVs are rich in phosphatidylserine, which provides a negatively charged surface that facilitates the binding of extrinsic and intrinsic tenase complexes [29]. Consequently, phosphatidylserine-rich platelet-derived EVs might fuse with monocytes and EVs derived from monocytes, increasing their procoagulant potential. 

One limitation of the present study is the small number of included patients with prostate cancer and DIC. However, DIC in prostate cancer is a rare condition, and so far, primarily retrospective analyses and clinical case reports have been published [4,5,6,7,8,9]. We included well-matched prostate cancer and healthy controls, determined routine and experimental parameters and performed in vitro co-culture experiments. 

Another limitation of our in vitro co-culture experiments needs to be mentioned: low TF activity and TF expression was observed on unstimulated PBMCs, which indicates slight activation during the isolation process and incubation [30,31]. This however was also observed by others [32] and most probably cannot be completely prevented.

In summary, our data indicate that elevated EV-TF activity plays an important role in prostate-cancer-related DIC and results from direct and indirect interactions between prostate cancer cells, monocytes, and platelets. 

## 5. Conclusions

Our data indicate that elevated EV-TF activity plays an important role in prostate-cancer-related DIC and results from direct and indirect interactions between prostate cancer cells, monocytes, and platelets. 

## Figures and Tables

**Table 1:** Clinical parameters of prostate cancer patients with disseminated intravascular coagulation (DIC) and prostate cancer patients without DIC.

Clinical Parameter, (Median, Range)	Prostate Cancer with DIC (*n* = 7)	Prostate Cancer without DIC (*n* = 10)	*p*-Value
Age, years	70 (53–73)	66 (49–69)	0.48
Gleason Score	7 (6–9)	7 (7–8)	0.88
Castration resistance Low dose anticoagulation	7 2	9 0	
Bleeding at inclusion	4	0	
Gastrointestinal	3	0	
Subarachnoidal	2	0	
Stroke at inclusion	1	0	

**Table 2:** Routine and experimental laboratory parameters of prostate cancer patients with DIC, age- and stage-matched prostate cancer patients without DIC, and age-matched healthy individuals.

Laboratory Parameter (Median, Range)	Prostate Cancer Patients with DIC (*n* = 7)	Prostate Cancer Patients without DIC (*n* = 10)	Healthy Controls (*n* = 10)	*p*-Value Patients with DIC Versus Patients without DIC	*p*-Value Patients with DIC Versus Healthy Controls	*p*-Value Patients without DIC Versus Healthy Controls
Routine parameter: Thrombocyte count, ×10^3^/μL	36 (24 to 119)	244.50 (177 to 528)	213.50 (172 to 326)	**0.001**	**0.001**	0.273
Leukocyte count, ×10^9^/L	5.69 (2.56 to 26.59)	7.36 (4.22 to 9.59)	5.95 (4.45 to 8.71)	0.040	0.354	0.131
Fibrinogen, %	99 (55 to 178)	379 (299 to 662)	331 (245 to 402)	**0.001**	**0.001**	0.130
Prothrombin time, %	60 (50 to 70)	105 (86 to 124)	98 (94 to 104)	**0.012**	**0.001**	0.462
aPTT, s	47.9 (40.0 to 65.20)	34.7 (24.5 to 40.6)	34.35 (29.70 to 36.6)	**0.001**	**0.001**	0.940
D-Dimer, ng/mL	44.83 (10.65 to 314.29)	0.53 (0.20 to 2.55)	0.39 (0.27 to 0.69)	**0.001**	**0.001**	0.362
PSA, ng/mL	264.7 (110.0 to 5000)	15 (0.5 to 680)	1.90 (0.97 to 2.81)	**0.008**	0.016	**0.043**
CRP, ng/mL	5.53 (1.17 to 53.20)	0.54 (0.07 to 4.20)	0.19 (0.03 to 0.53)	**0.007**	**0.001**	**0.050**
Experimental parameters:						
EV-TF activity, pg/mL	11.40 (4.34 to 27.06)	0.09 (0.00 to 0.30)	0.18 (0.09 to 0.54)	**0.001**	**0.001**	0.096
Fibrin clot formation time, s	346 (244.3 to 775)	948 (704 to 1312)	900 (583 to 1067)	**0.001**	**0.001**	0.496
Fibrin clot formation time + anti-TF antibody, s	920 s (725.5 to 1177)	975.5 (393 to 1640)	809 (591 to 1187)	0.669	0.161	0.104

>Bold *p*-values indicate statistical significance after Bonferroni correction (*p* < 0.01667). EV-TF—extracellular-vesicle-associated tissue factor.

## Data Availability

The data presented in this study are available on request from the corresponding author.
